# History of HPV in HPV-positive elderly women

**DOI:** 10.1016/j.eurox.2024.100297

**Published:** 2024-03-07

**Authors:** Ruth S. Hermansson, Gabriella Lillsunde-Larsson, Gisela Helenius, Mats G. Karlsson, Malin Kaliff, Matts Olovsson, Annika K. Lindström

**Affiliations:** a Department of Women’s and Children’s Health, Uppsala University, Uppsala, Sweden; b Department of Oncology, Faculty of Medicine and Health, Örebro University, Örebro, Sweden; c Department of Laboratory Medicine, Faculty of Medicine and Health, Örebro University, Örebro, Sweden; d School of Health Sciences, Örebro University, Örebro, Sweden

**Keywords:** HPV, Natural history, Cervical dysplasia, Women, Elderly

## Abstract

**Background:**

The aim of this study was to examine the natural course of HPV infection in women of 60 years and older who were HPV positive at inclusion, and any association between HPV positivity in historical samples and dysplasia outcome.

**Methods:**

Eighty-nine women aged 60–82 years, who tested positive for HPV between 2012 and 2016 were included. Sampling for cytology and/or histology was also performed. HPV genotyping was carried out on archived material back to 1999.

**Results:**

Of the 89 HPV-positive women 16 had HSIL, 34 had LSIL and 39 were benign at inclusion. Of the women with HSIL, 50.0% had the same HPV type in the archive samples, 12.5% had another type, and 37.5% were HPV negative. Among the 34 women with LSIL, 47.1% had the same HPV type in archive samples, 5.8% had another type, and 47.1% were HPV negative. Of the 39 women without dysplasia at inclusion, 25.6% had the same HPV type in archive samples, 5.1% had another HPV type and 69.2% were HPV negative.

**Conclusion:**

Surprisingly few of the elderly women thus seem to have a history with the same or any HPV infection the years before being diagnosed with an HPV infection and dysplasia. The significance of an HPV infection for dysplasia development in elderly women is still not fully understood.

## 1 Introduction

The primary cause of invasive cervical cancer and precancerous cervical lesions is a persistent infection with oncogenic types of human papillomavirus (HPV) [1], [2], [3]. In young women, the majority of HPV infections are transient [1]. The prevalence of HPV varies geographically, in population subgroups and depend on age [4], [5]. It is estimated to be around 11% throughout all age groups, but considerably higher among women aged 20–24 years, and decreasing with age [6], [7]. In some populations, a second peak has been observed among women 61–70 [8]. In elderly women, an HPV prevalence of around 4% has been reported [9], [10].

There is still no consensus concerning the definition of a persistent HPV infection. Several studies have shown that persistence of ∼ 6 months is associated with a strong summative relative risk as regards the association between HPV and precancerous lesions [11], [12], [13]. Persistence also varies with age, and some studies have shown a lower proportion of persistence in younger rather than older women [3], [14]. Few studies have compared HPV persistence, as monitored by an HPV genotyping approach, with a presence-absence HPV test over time [14], [15], [16]. A limitation is that most of these studies are on young women. It is uncertain whether an HPV infection that becomes undetectable on repeat testing has truly cleared. It is also impossible to know how long a woman, with a first positive test, has been infected prior to sampling. This may reduce the effectiveness of a single HPV test in terms of specificity for the prediction of a possible progressive disease.

Cervical cancer in women above the age of 65 is usually discovered at advanced stages and the prognosis is poor [17]. Knowledge regarding HPV biology in older women is scarce. To reduce the incidence of cervical cancer in this group of women it is necessary to gain knowledge of the prevalence, persistence, and latency of an HPV infection.

The primary aim of this work was to study the natural course of an HPV infection in older women by mapping present and historical HPV infections. As a secondary aim, we investigated any association between HPV positivity in historical samples and dysplasia.

## 2 Materials and methods

### 2.1 Sample collection

**Fig. 1:**
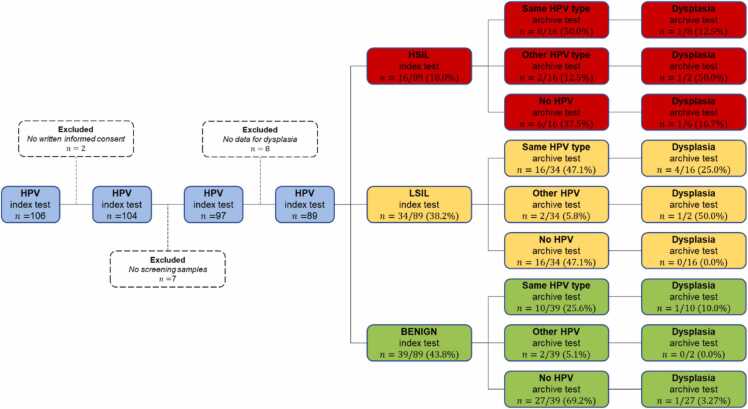
Flow chart showing study design, HPV, and dysplasia occurrence.

This retrospective, longitudinal, and descriptive study was based on 106 women aged 60–82 years, in Dalarna County, Sweden. Written informed consent was obtained from women who agreed to participate in the study. They had an HPV test (index test) as part of a gynecological examination, or by self-sampling, between May 2012 and November 2016. Two women did not give written informed consent and were excluded. Of the remaining 104 women, 97 women had archived cytology screening samples. At the time of the HPV index test, a clinical examination, including sampling for cytology and/or histology, was carried out on 89 women (Fig. 1). Cytology was conducted for all women and histology for 62 of the 89 women. All cytology and histology samples including screening samples of cytology were evaluated at the Department of Pathology and Cytology, Falun County Hospital, Falun, Sweden. The index HPV test was performed with HPVir [18]. HPV genotyping of archive samples was performed with Anyplex™ II HPV28 [19].

### 2.2 HPV genotyping on index sample using HPVIR

A sample of vaginal fluid was applied to an indicating FTA elute micro card™ (art. no WB129308, GE Healthcare, Longwood Dr, Cardiff CF14 7YT, UK). DNA was extracted, as described earlier, and the HPV test was performed using the real-time PCR assay HPVIR [18], [20]. The test detects and quantifies HPV 16, 18, 31, 33, 35, 39, 45, 51, 52, 56, 58, and 59 and provides single genotype information for all types except HPV18/45 and HPV33/52/58, which are detected together as two groups. The limit of detection (LOD) for HPVIR is 10 HPV copies per PCR. For a sample to contain a sufficient amount of material for the HPV test to be informative, a threshold of 10 copies of the nuclear single copy gene per PCR is used [18].

### 2.3 DNA extraction and HPV genotyping for archived samples

To validate the DNA extraction and HPV genotyping method to be used for archived cytology slides, a pilot study was conducted. For the pilot study, every tenth woman in the cohort was selected, n = 10.

DNA was extracted from cervical cytology slides. The cervical cytology slides were incubated in Xylene to enable the removal of the cover glass. The slides were each washed in 99.5%, 95%, and 70% ethanol and were left to air dry, after which the cells were dissolved in 180 µl ATL (Qiagen, Hilden, Germany) and transferred to a 2 ml sample tube RB (Qiagen). In the pilot study, DNA from the cells was extracted with the QIAamp DNA Mini kit (Qiagen) according to the instructions from the company using the QIAcube extraction platform (Qiagen). For the remaining subjects, the QIAamp DNA FFPE tissue kit (Qiagen) was used on the same platform.

HPV genotyping was performed with Anyplex™ II HPV28 (Seegene, Seoul, Korea), which detects 28 genotypes (HPV6, 11, 16, 18, 26, 31, 33, 35, 39, 40, 42, 43, 44, 45, 51, 52,53, 54, 56, 58, 59, 61, 66, 68, 69, 70, 73, and 82) and the human gene Beta-globin (*HBB).* A negative sample was determined to be negative if the control gene *HBB* was present at 40 cycles. Samples without a control gene result or HPV result were defined as invalid. The method has previously been validated on archival formalin fixed paraffin embedded (FFPE) material [19], [21]. DNA can be successfully extracted from an archived FFPE block stored over several years [22].

### 2.4 Cytology

Archived samples were obtained from the Department of Pathology and Cytology, Falun County Hospital, Falun, Sweden. We found 473 archive cytology samples from 89 women. On average 5.3 (median 4) samples for every study participant were analyzed (range 1–16). The samples were retrieved back in 1999.

A PAP-smear on glass was used until 2012 and liquid-based cytology (LBC) from 2013. All LBC specimens were screened by cytotechnicians, and those considered abnormal were reviewed by a surgical pathologist. For LBC, the Thin Prep® Pap Test was used. The cervical smear was collected with a plastic spatula and a cytobrush. LBC specimens were placed in PreserveCyt solution and processed in the Thin Prep 5000-processor (HologicCytyc Corporation, Boxborough, Mass) [23].

During the time period, the terminology for the classification of cytopathology was changed to The Bethesda System (TBS), with the classification low-grade intraepithelial lesion (LSIL) and high-grade intraepithelial lesion (HSIL)[24]. All the results have been presented in the Bethesda system.

The study was approved by the Swedish Ethical Review Authority (Dnr: 2016/441, 2020–06478).

### 2.5 Statistical analyses

For descriptive statistical analysis, Excel Microsoft Office Professional Plus 2016 was used.

## 3 Results

### 3.1 Index samples

At inclusion (index sample) all 12 HPV types detected by HPVIR were found. The most common HPV type was HPV 16 (28/89). Of the 89 HPV-positive women 16 had HSIL, 34 had LSIL and 39 were benign according to cytology and/or histology. Cytology alone was benign for 85.4% (76/89) of the women and 14.6% (13/89) had dysplasia (4 HSIL, 7 LSIL and 1 glandular cell atypia).

### 3.2 Archive samples

For all 89 women a total of 473 retrieved archive samples of cytology were analyzed. One or multiple HPV types were detected in 26.8% of the samples (127/473). Of the 473 cytology samples, 93.7% (443/473) were benign and 5.5% (26/473) showed some type of dysplasia (20 LSIL, 3 glandular cell atypia and 3 HSIL). Four of them were not assessable.

Of all 89 women 45.0% (40/89) were also HPV positive in one or more of the archive samples. Of these 40 women 85.0% (34/40) had the same HPV type in the historical samples, while 15.0% (6/40) had another HPV type. HPV could not be detected in 55.0% (49/89) of the women’s archive samples. HPV 16 was also the most frequent HPV type in the archive samples.

### 3.3 Screening history of women with dysplasia in index-sample

Of the 50 women with HSIL and LSIL 48% (24/50) had the same HPV type in archive samples, 8% (4/50) had another type, and 44% (22/50) were HPV negative.

#### 3.3.1 Screening history in women with HSIL in index-sample

**Fig. 2:**
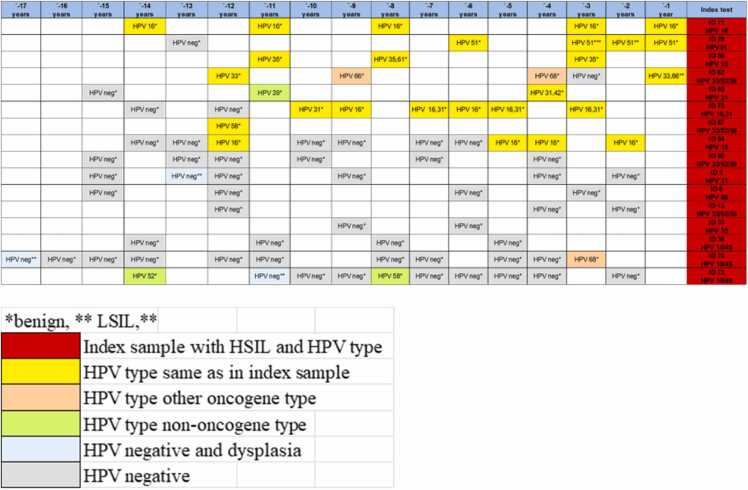
History of HPV and dysplasia in 16 women with HSIL in index sample. *benign, * *low grade squamous intraepithelial lesion (LSIL), * **high grade squamous intraepithelial lesion (HSIL).

The 16 women with HSIL in the index sample, had between 2 and 11 archived cytology samples available for analysis (average 5.8). Of the 16 women with HSIL, 50.0% (8/16) had the same HPV type in archive samples, 12.5% (2/16) had another type and 37.5% (6/16) were HPV negative. Of the HPV-negative women, 75.0% (12/16) did not have any detectable dysplasia in the archive samples while 25.0% (4/16) had dysplasia (2 LSIL and 2 HSIL) in their archive samples. For 7 of the 8 women with concordant HPV type in HSIL index- and screening samples, screening samples were HPV positive 6 years or more before the index sample, and in 6 of them 10 years or more. For women with HPV-negative archive samples, all women had at least 2 HPV-negative samples before the HPV-positive HSIL index test. In two cases, six HPV-negative samples (15 years earlier) proceeded the HPV-positive index test (Fig. 1, Fig. 2).

#### 3.3.2 Screening history of women with LSIL in index sample

**Fig. 3:**
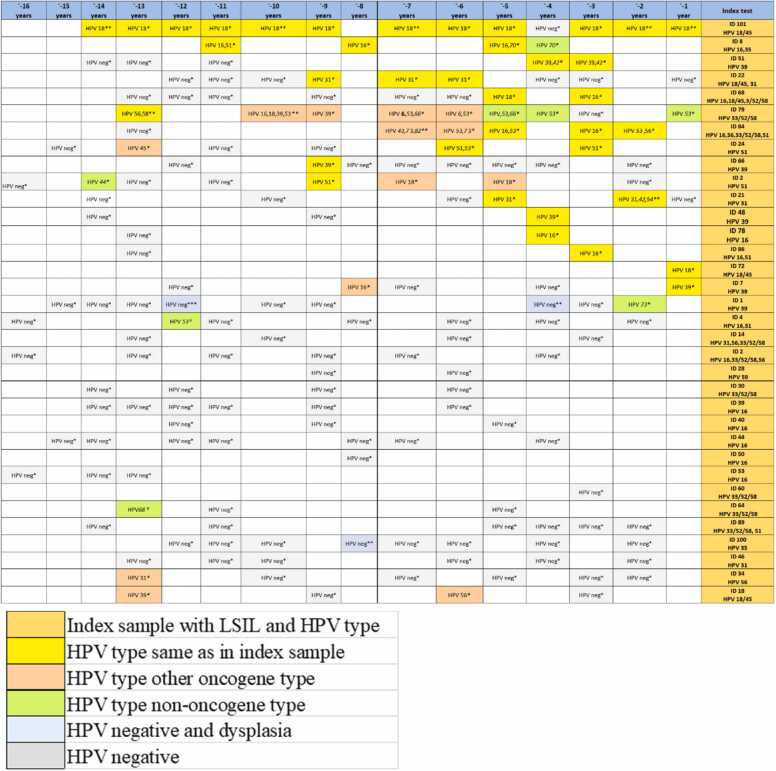
History of HPV and dysplasia in 35 women with LSIL in index sample. *benign, * *low grade squamous intraepithelial lesion (LSIL), * **high grade squamous intraepithelial lesion (HSIL).

Among the 34 women with LSIL, 47.1% (16/34) had the same HPV type in archive samples, 5.8% (2/34) had another type, and 47.1% (16/34) were HPV negative. Of the HPV-negative women, 85.3% (29/34) did not have any detectable dysplasia in the archive samples while 14.7% (5/34) had dysplasia (1 HSIL, 4 LSIL, 1 glandular cell atypia) (Fig. 1, Fig. 3).

### 3.4 Screening history of women with benign index-sample

**Fig. 4:**
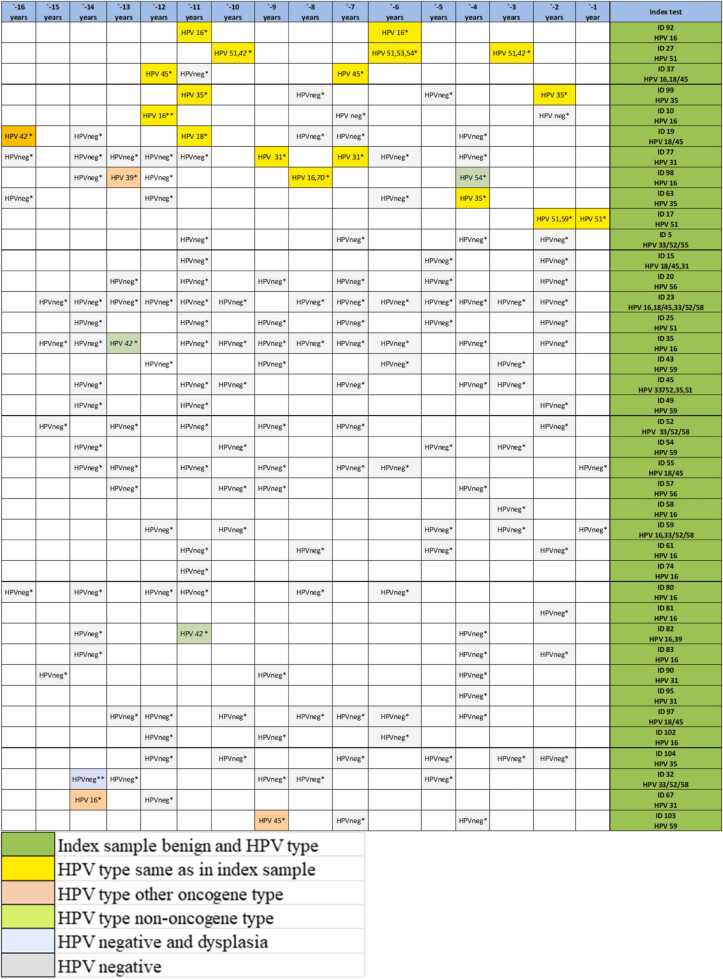
History of HPV and dysplasia in 35 women without dysplasia in index sample. *benign, * *low grade squamous intraepithelial lesion (LSIL), * *high grade squamous intraepithelial lesion (HSIL).

Of the 39 women with benign cytology at inclusion, 25.6% (10/39) had the same HPV type in archive samples, 5.1% (2/39) had another HPV type and 69.2% (27/39) were HPV negative. Only two of these 39 women had dysplasia in an archive sample (Fig. 1, Fig. 4).

## 4 Discussion

There are few studies on the significance of an HPV infection and precursor lesions in women above screening age [9], [25], [26], [27], [28]. Grainge and co-workers used archive smears to evaluate screening history for older women (>50) compared to younger women, and showed a high risk of HPV acquisition in these women [29]. Other studies have also evaluated the usefulness of cervical smears for HPV testing with good results [30], [31]. To our knowledge, there are no studies published about the natural history of an HPV infection and the risk for cervical dysplasia and cancer in elderly women.

Our results show that HPV-positive women with HSIL and LSIL, had HPV of the same type in historical samples as in the index sample in only about half of the cases. We cannot determine whether women with dysplasia who did not have HPV or other HPV in archived samples had a new infection or whether there may be latent undetectable infections in archived samples. In addition, only 25% of those with HSIL and 15% of those with LSIL, in their index sample had dysplasia in historical samples. Few women in this study had dysplasia in archived samples and this is consistent with what we know about screening with cytology having low sensitivity in post-menopausal women [9], [32], [33].

It is interesting to note that quite some of the women with many years of HPV positivity did not develop any dysplasia. This is well known in younger women and might of course also be the case in elderly women, probably reflecting health status, absence of immunosuppressive medication, etc. On the other hand, there was also a group of women with consistently negative historical HPV tests and yet they had HSIL or LSIL in their index sample. Some of these cases can most probably be explained by rather rapid progress from normality to LSIL or HSIL, perhaps due to aging, bad general health, impaired immunocompetence, etc. Another explanation could be that some of the HPV analyses of historical cytological samples were false negatives.

Whether the heterogeneity of the outcome of these presumably persistent HPV infections is a result of methodological challenges or biological differences, can in this study setting only be hypothesized. Low tissue amount and old archive material is a challenge and could have resulted, despite sensitive extraction, detection, and genotyping methods, in false negatives. However, the varying results in the clinical outcome of persistent infection in the present cohort could very well be affected by parameters like host immunocompetence and differences in viral oncogenic competency. Factors that would be of interest to study in the future are the differences in viral characteristics such as viral load, integration- and methylation status in elderly women with persistent infections, with varying clinical outcomes.

There are several limitations to the study. An obvious weakness is the HPV analyses of the historical cytology samples with the risk of false negatives. The Anyplex II HPV28 has been validated on FFPE samples and includes a human control gene to verify DNA suitability for analysis and any PCR inhibition in the multiplex PCR. The method has been shown to have a detection limit of 50 copies/reaction, and has been used in clinical settings for genotyping of HPV but has also been successful in research studies on historical FFPE cohorts [19], [34]. It is however not validated on archived PAP smears. Archival smears may differ in cell amount and viral nucleic acid content and also in how they are affected by long-term storage. False negatives may pose a risk, especially in samples holding a low viral load below the detection limit, i.e. latency. Other limitations include a rather low number of cases, which introduces some uncertainty about proportions and the fact that the historical samples in some cases are rather few and do not go so far back in time. There was a lack of demographic data such as smoking, general health, sexual activity, number of partners, diseases, immunodeficiency, medical treatments, etc.

There are some strengths of the study. One is that all the index samples were analyzed with the same method, HPVir, which is a validated HPV test [35], [36]. Another is that all archive samples were analyzed with the same method, Anyplex, which is also a validated HPV test [36]. A third strength is that all the women included were from the same region in Sweden and they were therefore examined using the same clinical protocol and the same pathology department. Also, it is unique to find historical samples going back sixteen years for these elderly women.

## 5 Conclusion

Surprisingly few of the elderly women seem to have a history with the same or any HPV infection the years before being diagnosed with an HPV infection and dysplasia. The significance of an HPV infection for dysplasia development in elderly women is thus still not fully understood.

## Funding

Open access funding provided by Uppsala University, Sweden. The Regional Research Council Uppsala -Orebro, Sweden, grant number RFR-644831 supported this work. The funders had no role in study design, data collection and analysis, decision to publish, or preparation of the manuscript.

## CRediT authorship contribution statement

**Matts Olovsson:** Writing – review & editing, Writing – original draft, Validation, Supervision, Methodology, Formal analysis. **Annika Kristina K Lindstrom:** Writing – review & editing, Writing – original draft, Visualization, Validation, Supervision, Software, Resources, Project administration, Methodology, Investigation, Funding acquisition, Formal analysis, Data curation, Conceptualization. **Malin Kaliff:** Writing – review & editing, Methodology, Investigation, Formal analysis, Data curation. **Ruth S Hermansson:** Writing – review & editing, Writing – original draft, Validation, Methodology, Investigation, Formal analysis, Data curation. **Gabriella Lillsunde-Larsson:** Writing – review & editing, Writing – original draft, Validation, Supervision, Resources, Methodology, Investigation, Formal analysis. **Gisela Helenius:** Writing – review & editing, Validation, Supervision, Methodology, Investigation, Formal analysis. **Mats G. Karlsson:** Writing – review & editing, Supervision, Methodology, Formal analysis, Conceptualization.

## Declaration of Competing Interest

None.

## References

[1] Ho G.Y., Bierman R., Beardsley L., Chang C.J., Burk R.D. (1998). Natural history of cervicovaginal papillomavirus infection in young women. N Engl J Med.

[2] Walboomers J.M., Jacobs M.V., Manos M.M., Bosch F.X., Kummer J.A., Shah K.V. (1999). Human papillomavirus is a necessary cause of invasive cervical cancer worldwide. J Pathol.

[3] Hildesheim A., Schiffman M.H., Gravitt P.E., Glass A.G., Greer C.E., Zhang T. (1994). Persistence of type-specific human papillomavirus infection among cytologically normal women. J Infect Dis.

[4] Franceschi S., Herrero R., Clifford G.M., Snijders P.J., Arslan A., Anh P.T. (2006). Variations in the age-specific curves of human papillomavirus prevalence in women worldwide. Int J Cancer.

[5] de Sanjose S., Diaz M., Castellsague X., Clifford G., Bruni L., Munoz N. (2007). Worldwide prevalence and genotype distribution of cervical human papillomavirus DNA in women with normal cytology: a meta-analysis. Lancet Infect Dis.

[6] Bosch F.X. (2003). Epidemiology of human papillomavirus infections: new options for cervical cancer prevention. Salud Publica Mex.

[7] Bruni L., Diaz M., Castellsague X., Ferrer E., Bosch F.X., de Sanjose S. (2010). Cervical human papillomavirus prevalence in 5 continents: meta-analysis of 1 million women with normal cytological findings. J Infect Dis.

[8] Yan X., Shen L., Xiao Y., Wang Q., Li F., Qian Y. (2021). Prevalence, characteristics, and distribution of HPV genotypes in women from Zhejiang Province, 2016-2020. Virol J.

[9] Hermansson R.S., Olovsson M., Hoxell E., Lindstrom A.K. (2018). HPV prevalence and HPV-related dysplasia in elderly women. PLoS One.

[10] Andersen B., Christensen B.S., Christensen J., Ejersbo D., Heje H.N., Jochumsen K.M. (2019). HPV-prevalence in elderly women in Denmark. Gynecol Oncol.

[11] Koshiol J., Lindsay L., Pimenta J.M., Poole C., Jenkins D., Smith J.S. (2008). Persistent human papillomavirus infection and cervical neoplasia: a systematic review and meta-analysis. Am J Epidemiol.

[12] Rositch A.F., Koshiol J., Hudgens M.G., Razzaghi H., Backes D.M., Pimenta J.M. (2013). Patterns of persistent genital human papillomavirus infection among women worldwide: a literature review and meta-analysis. Int J Cancer.

[13] Zhao M., Zhou D., Zhang M., Kang P., Cui M., Zhu L. (2023). Characteristic of persistent human papillomavirus infection in women worldwide: a meta-analysis. PeerJ.

[14] Munoz N., Hernandez-Suarez G., Mendez F., Molano M., Posso H., Moreno V. (2009). Persistence of HPV infection and risk of high-grade cervical intraepithelial neoplasia in a cohort of Colombian women. Br J Cancer.

[15] Bory J.P., Cucherousset J., Lorenzato M., Gabriel R., Quereux C., Birembaut P. (2002). Recurrent human papillomavirus infection detected with the hybrid capture II assay selects women with normal cervical smears at risk for developing high grade cervical lesions: a longitudinal study of 3,091 women. Int J Cancer.

[16] Dalstein V., Riethmuller D., Pretet J.L., Le Bail Carval K., Sautiere J.L., Carbillet J.P. (2003). Persistence and load of high-risk HPV are predictors for development of high-grade cervical lesions: a longitudinal French cohort study. Int J Cancer.

[17] Darlin L., Borgfeldt C., Widen E., Kannisto P. (2014). Elderly women above screening age diagnosed with cervical cancer have a worse prognosis. Anticancer Res.

[18] Moberg M., Gustavsson I., Gyllensten U. (2003). Real-time PCR-based system for simultaneous quantification of human papillomavirus types associated with high risk of cervical cancer. J Clin Microbiol.

[19] Lillsunde Larsson G., Carlsson J., Karlsson M.G., Helenius G. (2015). Evaluation of HPV genotyping assays for archival clinical samples. J Mol Diagn.

[20] Gustavsson I., Juko-Pecirep I., Backlund I., Wilander E., Gyllensten U. (2009). Comparison between the Hybrid Capture 2 and the hpVIR real-time PCR for detection of human papillomavirus in women with ASCUS or low grade dysplasia. J Clin Virol.

[21] Mathieson W., Thomas G.A. (2020). Why Formalin-fixed, Paraffin-embedded Biospecimens Must Be Used in Genomic Medicine: An Evidence-based Review and Conclusion. J Histochem Cytochem.

[22] Kokkat T.J., Patel M.S., McGarvey D., LiVolsi V.A., Baloch Z.W. (2013). Archived formalin-fixed paraffin-embedded (FFPE) blocks: A valuable underexploited resource for extraction of DNA, RNA, and protein. Biopreserv Biobank.

[23] Carpenter A.B., Davey D.D. (1999). ThinPrep Pap Test: performance and biopsy follow-up in a university hospital. Cancer.

[24] Soloman D. (1989). The 1988 Bethesda system for reporting cervical/vaginal cytologic diagnoses: developed and approved at the national cancer institute workshop in Bethesda. Diagn Cytopathol.

[25] Gravitt P.E., Winer R.L. (2017). Natural History of HPV Infection across the Lifespan: role of Viral Latency. Viruses.

[26] de Sanjose S., Brotons M., Pavon M.A. (2018). The natural history of human papillomavirus infection. Best Pr Res Clin Obstet Gynaecol.

[27] Bergengren L., Karlsson M.G., Helenius G. (2020). Prevalence of HPV and pathological changes among women 70 years of age, 10 years after exclusion from the Swedish cervical cancer screening program. Cancer Causes Control.

[28] Lanner L., Lindstrom A.K. (2020). Incidence of HPV and HPV related dysplasia in elderly women in Sweden. PLoS One.

[29] Grainge M.J., Seth R., Guo L., Neal K.R., Coupland C., Vryenhoef P. (2005). Cervical human papillomavirus screening among older women. Emerg Infect Dis.

[30] Tabrizi S.N., Taylor N., McCullough M.J., Phillips G., Wark J., Gertig D. (2010). Human papillomavirus genotype detection from archival papanicolaou-stained cervical tests. Cancer Cytopathol.

[31] Jacobs M.V., Zielinski D., Meijer C.J., Pol R.P., Voorhorst F.J., de Schipper F.A. (2000). A simplified and reliable HPV testing of archival Papanicolaou-stained cervical smears: application to cervical smears from cancer patients starting with cytologically normal smears. Br J Cancer.

[32] Lindstrom A.K., Hermansson R.S., Gustavsson I., Hedlund Lindberg J., Gyllensten U., Olovsson M. (2018). Cervical dysplasia in elderly women performing repeated self-sampling for HPV testing. PLoS One.

[33] Gustafsson L., Sparen P., Gustafsson M., Pettersson B., Wilander E., Bergstrom R. (1995). Low efficiency of cytologic screening for cancer in situ of the cervix in older women. Int J Cancer.

[34] Kaliff M., Sorbe B., Mordhorst L.B., Helenius G., Karlsson M.G., Lillsunde-Larsson G. (2018). Findings of multiple HPV genotypes in cervical carcinoma are associated with poor cancer-specific survival in a Swedish cohort of cervical cancer primarily treated with radiotherapy. Oncotarget.

[35] Gustavsson I., Aarnio R., Myrnas M., Hedlund-Lindberg J., Taku O., Meiring T. (2019). Clinical validation of the HPVIR high-risk HPV test on cervical samples according to the international guidelines for human papillomavirus DNA test requirements for cervical cancer screening. Virol J.

[36] Arbyn M., Simon M., Peeters E., Xu L., Meijer C., Berkhof J. (2021). 2020 list of human papillomavirus assays suitable for primary cervical cancer screening. Clin Microbiol Infect.

